# Surgical Methods and Devices for Atrial Fibrillation

**DOI:** 10.31083/RCM26841

**Published:** 2025-04-23

**Authors:** Yalu Yu, Qin Jiang

**Affiliations:** ^1^School of Medicine, University of Electronic Science and Technology of China, 610054 Chengdu, Sichuan, China; ^2^Department of Cardiac Surgery, Sichuan Provincial People’s Hospital, Affiliated Hospital of University of Electronic Science and Technology, 610072 Chengdu, Sichuan, China; ^3^Ultrasound in Cardiac Electrophysiology and Biomechanics Key Laboratory of Sichuan Province, Sichuan Provincial People’s Hospital, University of Electronic Science and Technology of China, 610072 Chengdu, Sichuan, China

**Keywords:** atrial fibrillation, Cox-Maze, left atrial appendage closure, surgical devices

## Abstract

As technology advances, surgical approaches for atrial fibrillation have diversified. Surgical treatments include Cox-Maze surgery, left atrial appendage occlusion, or closure using a clip. Cox-Maze surgery removes excessive cardiac electrical conduction pathways, ensures electrical signals propagate exclusively through the predetermined maze channel and restores normal heart rhythm. Left atrial appendage closure reduces the risk of long-term disability or death caused by left atrial appendage thromboembolism in patients with atrial fibrillation. These devices are constantly being refined, including bipolar radiofrequency clamps (monopolar or bipolar radiofrequency), left atrial appendage closure devices (external excision using staplers, internal ligation with biomatrix patch occlusion, external device placement with the AtriClip and Endoloop ligature). In addition to surgical interventions, surgical biomaterial materials with biocompatibility and electrical conductivity have emerged in the basic research phase of atrial fibrillation treatment. This review delineates the primary surgical techniques, emphasizing their safety and efficacy in treating atrial fibrillation. An introduction to commonly used surgical equipment is provided as a reference for the clinical management of atrial fibrillation.

## 1. Introduction

Atrial fibrillation (AF) is the most frequent arrhythmia, and is more commonly seen in older patient [1]. The frequency of AF has doubled within the last three decades, and projections indicate that by 2030, the prevalence in the United States will surge to 12.0 million, while in European nations, it is anticipated to increase to 17.9 million by 2060 [2]. The mechanisms for the development of AF include stretch-induced fibrosis in the atrial tissue [3], greater volume of epicardial adipose tissue [4], persistent inflammation due to the oxidative stress response [5], AF-dependent ion channel remodeling and the rotor machinery [6], vagal hyperactivity and inherited genes associated with AF [7]. The consequences of AF are an elevated risk of stroke and heart failure [8], which exacerbate existing co-morbidities such as renal insufficiency [9], resulting in increased medical costs and hospitalizations [10]. These factors have the potential to significantly impair the quality of life for patients, leading to increased morbidity and mortality [11]. In recent years, guidelines for the management of atrial fibrillation have undergone continuous updates to optimize treatment [12]. Despite the fact that catheter ablation plays an important role in the maintenance of sinus rhythm [13], up to 30% of patients with persistent AF experience a relapse within a year [14]. Surgical procedures and hybrid ablation have shown more promising results, particularly in patients with more advanced disease [15].

## 2. Literature Review

### 2.1 Surgical Procedures and Therapeutic Devices 

Current surgical procedures that have achieved clinical success are primarily focused on the eradication of AF and the prevention of AF-related cerebrovascular accidents, such as stroke (ischemic or hemorrhagic) and systemic embolism [16, 17]. Surgery for AF aims to restore sinus rhythm, improve hemodynamics, and reduce the risk of thromboembolism and stroke, while reducing the need for repeat surgery [18]. As medical technology advances, surgical ablation equipment for treating atrial fibrillation has experienced significant innovations in recent years.

#### 2.1.1 Cox-Maze Procedure and Development

The cut and sew Cox-Maze I procedure was developed in 1987 by Dr. James L. Cox and colleagues [19]. The Cox-Maze I procedure progressed from Maze II to Maze III and is considered to be a very successful procedure to terminate AF [20]. This technique aims to create barriers based on the reentry circuit of macro-scale AF through maze incisions and sutures by in both atria [21]. The Cox-Maze III procedure is considered the gold standard for treating AF, but it is complex and difficult to perform [22]. Therefore, the Cox-Maze IV procedure, which is characterized by its simplicity and speed, was introduced in 2002 [23]. In a subsequent study spanning 15 years (from May 2003 to March 2018), among 853 patients who underwent the Cox-Maze IV procedure, the incidence of initial atrial fibrillation recurrence was 11%, 23%, and 35% at different time points, demonstrating the long-term effectiveness of the Cox-Maze IV procedure in sustaining normal sinus rhythm compared to catheter ablation and other surgical methods for treating atrial fibrillation [24]. The Cox-Maze IV procedure also facilitates long-term restoration of sinus rhythm in patients with rheumatic or degenerative mitral valve disease (MVD). Although the duration of preoperative AF tends to be longer in patients with rheumatic MVD, patients with rheumatic MVD also benefit from the Cox-Maze IV procedure [25].

**Fig. 1.**
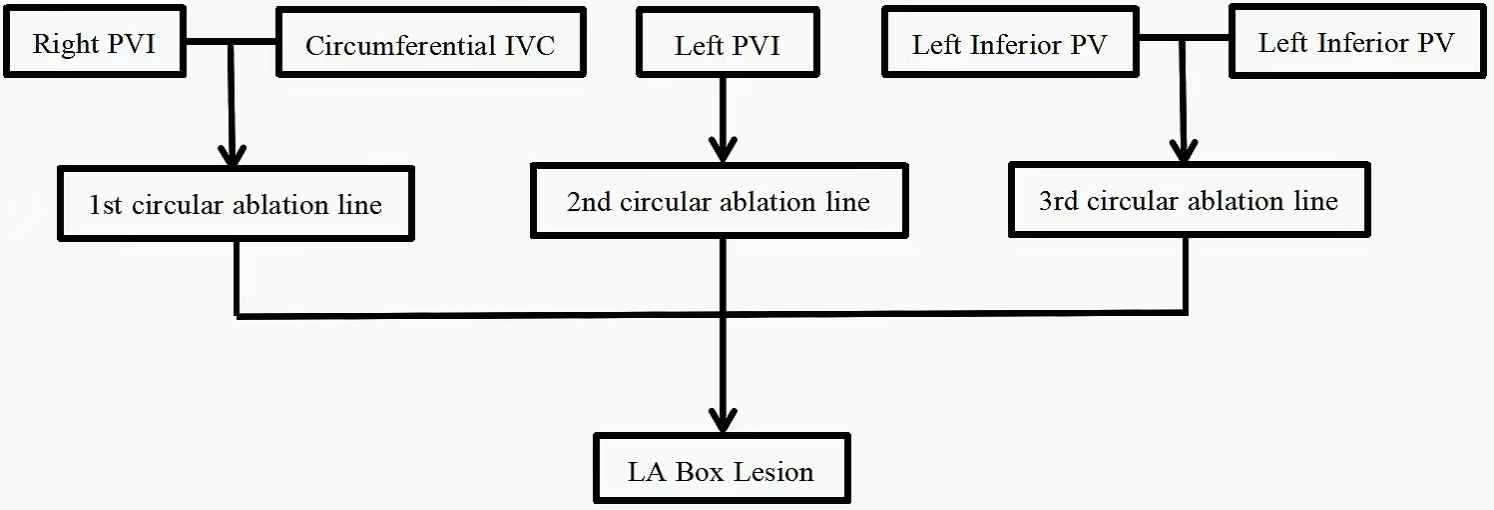
**The three ablation lines of Mei mini-maze procedure**. PVI, pulmonary vein isolation; IVC, inferior vena cava; PV, pulmonary vein; LA, left atrium.

Wolf mini-maze is a minimally invasive maze procedure that takes approximately 90 minutes to complete, and is associated with a low incidence of cerebral microemboli during the procedure [26]. The principle is to separate the left atrium (LA), isolate the pulmonary veins (PVs), and clamp the left atrial appendage (LAA) [27]. Although the Wolf mini-maze procedure was linked to diminished surgical trauma, it still necessitated a bilateral thoracotomy. In addition, the ablation line that connects the right pulmonary vein isolation (PVI) to the left PVI could not be established using bipolar ablation forceps [28]. Consequently, a novel video-assisted mini-maze technique has been developed, employing a unilateral (left thoracic) thoracoscopic approach using only three ports and three ablation lines (Fig. 1), known as the Mei mini-maze procedure [29]. During total thoracoscopy, monopolar ablation forceps are typically employed [30]. The total thoracoscopy approach is beneficial in reducing the inflammatory response associated with surgical procedures [31]. However, total thoracoscopic surgery is more difficult to perform, requires multimodality cardiovascular imaging guidance [32], and collaboration from a multi-disciplinary working group including surgeons, anesthesiologists, perfusionists, echocardiographers and nurses [33].

The original maze procedure was developed to combine new ablation techniques with minimally invasive methods [34]. In the last ten years, there has been a significant increase in the annual number of standalone ablation procedures [35]. Minimally invasive ablation for atrial fibrillation has been demonstrated to be relatively safe and effective [36]. Studies have demonstrated that this surgical procedure can significantly reduce the incidence of AF at both one and three years postop [37]. In a study of 133 patients undergoing a right thoracotomy measuring 5 cm under cardiopulmonary bypass, 73 discontinued antiarrhythmic drugs after a single intervention, there was 1 stroke, 13 patients had atrial arrhythmias, 15 patients required a cardioversion [38]. To enhance the perioperative safety and long-term efficacy of minimally invasive Cox-Maze surgery, surgical techniques are continuously being refined.

**Fig. 2.**
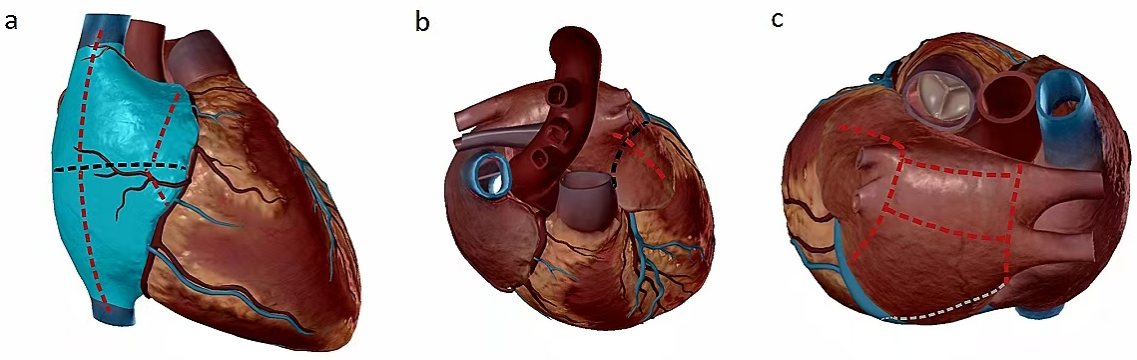
**Cox-Maze IV ablation lesions (black line: incision; red line: ablation lines caused by ablation clamp; gray line: ablation lines caused by melting pen)**. (a) The incision on the right atrial free wall was executed utilizing ablation clamps, specifically for the superior and inferior vena cava; dissection of the right atrioventricular groove was carried out, followed by clamp ablation via a vertical incision; the right atrial auricle was clamped, extending from the atrial free wall incision to the auricle itself. (b) The left atrium was meticulously excised and ligated, followed by the ablation of the connection between the left atrium and the left superior pulmonary vein utilizing specialized ablation clamps. (c) The procedure involves the isolation of the right and left pulmonary veins using an ablation clamp, followed by the ablation of the left pulmonary vein with the ablation clamp, commencing from the left towards the left pulmonary vein. An ablation is performed from the lower right pulmonary vein to the lower left pulmonary vein. This is succeeded by the ablation of the left atrial isthmus, utilizing bipolar forceps for coagulation. The process further entails the coagulation of the pulmonary veins and supplementary ablation within the epicardial region of the left atrium, along the coronary sinus route for left atrial ablation.

The surgical approach for AF also employs a minimally invasive method for managing the left atrial appendage, coupled with surgical ablation. This technique involves a comprehensive endoscopic surgical procedure conducted without the need for cardiopulmonary bypass [39]. An isolator transpolar pen is used to form the trigonum line after isolation of the left and right pulmonary veins. The multifunctional linear pen creates the roof line and floor line to constitute the box lesions. The primary endpoint of surgery is restoration of sinus rhythm and to create bidirectional block using the box lesion [40, 41]. The procedure is also known as the Wolf-Ohtsuka method or the total thoracoscopic maze (TT-maze). The port placement device requires only a single incision, allowing for more rapid patient recovery [42]. Although it has a smaller incision than the mini-maze procedure, the operative time is doubled, and there is an increased risk for complications including pleural effusions and a pneumothorax [43]. In an analysis of 14 studies published between 2011 and 2016, the TT maze showed similar efficacy to Cox Maze IV surgery at mid-term follow-up, with a low incidence of major complications (3%) and an overall incidence of stroke of 0.34% [44]. After six months to one year of follow-up, there was an 80% to 90% chance of remaining free from atrial fibrillation [45, 46]. A modified procedure employing a combined total thoracoscopic maze technique was also utilized. In this approach, a bilateral thoracoscopy was used to inspect the atrial epicardial lesions, followed by endocardial catheter mapping and ablation three months later [47]. In a follow-up of 20 patients undergoing this procedure, the absence of AF was 100% after one year [48]. Therefore, the modified thoracoscopic maze procedure exhibits excellent safety and effectiveness in creating many of the Cox-Maze IV lesion sets (Fig. 2).

#### 2.1.2 Cox-Maze Procedure Corresponding Devices

With significant advances in Cox-Maze surgery and related minimally invasive methods, new energy technologies, including radio frequency, cryoablation, microwave, and high-intensity focused ultrasound (HIFU), have gradually supplanted traditional ablative methods [49, 50, 51]. At present, the ablation equipment commonly employed primarily encompasses the double-pulse radiofrequency (RF) clamp and cryoablation devices. The efficacy of monopolar radiofrequency ablation remains a subject of debate. Studies have shown that bipolar radiofrequency (BPRF) ablation is more effective and more convenient to use than monopolar radiofrequency ablation (RFA) [52, 53]. However, studies have also shown that monopolar RFA has a better postoperative quality of life, and that flushing monopolar RFA can also treat atrial fibrillation in adult patients with congenital heart defects [54, 55]. Although monopolar radiofrequency ablation ensures the continuity of the ablation line, the transmurality is poor, achieving only 33.3% in animal experiments [56, 57]. Cryoablation causes the temperature of the device to rise, which in turn leads to a reduction in the efficiency of the ablation process [58]. Therefore, it is primarily utilized for endocardial ablation during cardiac arrest, while epicardial ablation beneath the heart is not recommended [59].

BPRF energy is delivered via a pair of pulsed electrodes, each measuring 1 mm in width and 5 cm in length, through the aperture of the clamp [60]. The configuration effectively mitigates lateral thermal diffusion, ensuring that the current density is meticulously localized within the tissue situated between the electrodes [61]. This facilitates the effortless accumulation of energy, which may result in sustained damage, mitigating the impact on the side wall or pericardial region [62]. The ablative efficacy can remain uniform across all types of the tissue, and such efficacy is possible even within denser and more recalcitrant tissues [63]. Within an interval of approximately 7 to 12 seconds, a transmural linear lesion was meticulously and dependably created in both atria, accomplishing electrical isolation of the pulmonary veins. This method, which avoids cardiopulmonary bypass, markedly shortens the duration of the surgical procedure [64]. It is imperative that bipolar radiofrequency ablation clamps are utilized under direct visualization, and they can be adapted for performance in a thoracoscopic manner [65]. The clamp is also subject to the influence of various elements during the ablation procedure, including atmospheric conditions, adipose tissue, cavity catheters, or electrodes, which may impact the transmission of energy and modulate the depth of ablation [66]. Consequently, it is imperative that the clamp maintains complete contact with the ablation zone [67]. In previous studies, it had been demonstrated that a BPRF clamp is an effective technique for the treatment of long-term AF in rheumatic valve disease [68, 69, 70]. BPRF systems are categorized into two distinct types: “non-irrigation” ablation forceps and an “irrigation” ablation device. The former are primarily AtriCure products (https://www.atricure.com/healthcare-professionals/therapies/concomitant-surgical-ablation/products) [71]. A prospective multicenter study involving 363 patients diagnosed with non-paroxysmal AF was conducted to evaluate the efficacy of the Cox-Maze IV procedure using AtriCure’s bipolar RF clamp. The results indicated that 80% of the patients were free of AF after one year, 78% after two years, and 76% after three years [24]. The latter are primarily Medtronic products (https://www.medtronic.com). The ablation of human atrial tissue using flushed BFRF clips does not consistently yield transmural lesions when performed only after one application. To markedly enhance the transmurality of the lesions, a dual ablation procedure is necessary [72]. A study was conducted comparing the effectiveness of non-irrigated and irrigated BPRF ablation products for the treatment of rheumatic valvular disease (Table 1) [73].

**Table 1.** **The comparison of non-irrigated and irrigated BPRF ablation products**.

BPRF ablation products	Non-irrigation	Irrigation
Difference	Straight clamp	Curved clamp
	Direct heating without saline irrigation	Heating with saline irrigation
	Easy to form eschar	Prevent to form eschar
Similarity	The RF time is positively correlated with the LA size	
	The SR recovery efficiency and safety are similar	

BPRF, bipolar radiofrequency; RF, radiofrequency; SR, sinus rate.

#### 2.1.3 Surgical Exclusion of the Left Atrial Appendage 

Patients with AF face a fivefold increased risk of stroke due to thromboembolism [74]. The current CHA_2_DS_2_-VASc scoring system is used to assess the risk of embolization in patients with AF [75]. Most of the strokes associated with AF are caused by cardiogenic thrombus derived from the LAA [76]. This is attributed to the fact that the LAA serves as a significant source of ectopic agitation and a primary location for the development of thrombosis in individuals afflicted with AF [77]. Studies had showed that 57% of thrombotic strokes in rheumatic AF cases are attributed to occurrences within the LAA [78]. This is due to decreased systemic blood flow due to damaged left-sided valves [79]. Study shows that 90% of thrombi in non-rheumatic AF are located in the LAA [80]. Current US and European guidelines has been updated, supporting surgical left atrial appendage isolation in all patients with atrial fibrillation during cardiac surgery, regardless of the presence of underlying valvular disease [75]. The guidelines suggest that for individuals with atrial fibrillation who are undergoing cardiac surgery, the closure of the left atrial appendage should be considered as an adjunct to oral anticoagulation therapy to reduce the risk of ischemic stroke and thromboembolism (moving from a Class IIb, level of evidence C recommendation to a Class I, level of evidence B). Due to the absence of high-quality clinical trials and definitive benefits, the guidelines persist in recommending left atrial occlusion for stroke prevention in atrial fibrillation, but the level of evidence has been downgraded (from B to C) [81]. The LAA resembles a pouch situated on the left-hand side of the heart; therefore the exclusion of LAA has been targeted to prevent thromboembolic complications [78]. The surgical exclusion of the LAA, frequently in conjunction with mitral valve surgery and coronary artery bypass grafting (CABG), or as part of the Cox-Maze procedure, has been performed for over six decades [82, 83, 84]. In a comparative study between different surgical techniques for left atrial exclusion in patients undergoing concomitant cardiac surgery, surgical exclusion techniques for the LAA include manual resection and oversewing of the LA stump with sutures, internal or epicardial suture ligation, as well as transepicardial left atrial appendage clipping and thoracoscopic LAA ligation [85]. Since the excised appendages has no thrombus at the base or at the stump, surgical resection of the LAA has emerged as the most effective occlusion technique, demonstrating the lowest rate of postoperative stroke, transient ischemic attacks (TIA) and late neurologic events [86, 87]. An intra-atrial resection procedure for the LAA, involving pulling the LAA into the LA with forceps, and closing the orifice with sutures directly from within the LA, ensured the complete removal of the LAA at its orifice [88]. The success rate of epicardium surgical resection using these suture techniques has been reported to be as high as 73% [89]. However, the epicardial resection of the appendage can occasionally pose challenges due to the necessity of cardiac repositioning. Although the LAA is the primary source of thromboemboli, it is also possible that additional sites of atrial thrombi may be present in patients with AF [90]. Surgical exclusion of the LAA may not completely eradicate the risk of thromboembolic complications in elderly patients with AF, heart failure, and/or severe left ventricular dysfunction, and large left atrial volume [91, 92].

The clinical data for preventing AF and stroke by excluding the LAA through ligation is robust [93]. Complete LAA ligation has been shown to be more effective than incomplete ligation in reducing late embolization and ischemic stroke during mitral valve replacement and off-pump coronary surgery [94, 95]. For patients diagnosed with non-paroxysmal AF, achieving no atrial arrhythmias at the 12-month mark, when compared solely to pulmonary vein isolation, represents a significant milestone [96]. The ligation of the LAA through endocardial suture necessitates the utilization of cardiopulmonary bypass and involves incisions in dome of the left atrium. This procedure inherently poses risks of bleeding and potential injury to the circumflex coronary artery [97]. Furthermore, endocardial suture ligation is incomplete in 10%–30% of patients [98]. Due to the frequent tearing of tissue, LAA residual flow is frequently seen on follow up imaging studies [99]. Endocardial ligation was initially conducted utilizing a simple suture ligation, but this approach was plagued by a high failure rate [100]. Most surgeons opt for a single-or double-layer endocardial suture exclusion, which is a more effective approach [101]. However, Katz *et al*. [102] demonstrated that the rate of failure associated with this technique remains as high as 36%. Therefore, innovative technologies have now been developed for internal ligation. A surgeon inverts the LAA into the LA and meticulously places a precisely locatable “compressive suture”, starting from the tip of the attachment and traversing its entire length to the base. Subsequently, the LAA is sutured in a methodical manner (purse string suture around the base of the LAA and a reinforce running suture), ensuring the compression suture is tightened appropriately and effectively bundled [103]. Another technique for the internal occlusion of the LAA is achieved using a biological matrix patch (CorMatrix ECM, Roswell, GA, USA) in the healthy left atrial endocardium [104]. Epicardial suture ligation can be accomplished without resorting to cardiopulmonary bypass (CPB) surgery and without the necessity of opening the LA [105]. This is appropriate for patients undergoing simultaneous cardiac surgery procedures (such as CABG and aortic valve surgery), those with a high risk for bleeding, requiring repeat cardiac surgery, or a failed percutaneous (transcatheter) exclusion procedure [106]. Epicardial ligation mitigates AF related arrhythmias through diminishing atrial dispersion [107]. Studies have demonstrated that epicardial closure leads to more advantageous hemodynamics compared to endocardial closure [26]. The initial epicardial ligation was accomplished utilizing a detachable snare loop (Endoloop), which was precisely positioned at the base of the LAA and subsequently contracted [108]. An implantable soft silicone occlusion fastener (TigerPaw II system) approved by the Food and Drug Administration (FDA) offered an alternative to manual suturing or staples with or without reinforcement [109]. The TigerPaw Pro has resulted in easier ostial access and deployment resulting in safe and efficient left atrial appendage occlusion (LAAO) procedures [110]. Another epicardial clip (AtriClip) can be placed under direct visualization and integrated with other open cardiac surgical procedures, to achieve ligation and electrical isolation [97]. In one study, 15 patients with continuous AF were assigned to an intraoperative LAAO using the TigerPaw System II (n = 8) or the AtriClip (n = 7) device. The Atri-Clip demonstrating safer epicardial LAAO during off-cardiopulmonary bypass CABG in patients with persistent AF [111]. The first report of a single incision, thoracoscopic epicardial left appendage ligation emerged in 2021, showcasing the practicality and safety of a novel approach utilizing an epicardial clip [112].

The creation of innovative percutaneous left atrial appendage occlusion (PLAAO) and transepicardial left atrial appendage clipping therapeutic approaches have sought to mitigate the consequences of AF embolization [113]. Studies showed that LAA isolation significantly reduced the incidence of perioperative stroke and mortality [114]. In patients with AF who have contraindications to surgery, LAA exclusion has shown perioperative safety, technical success, and no incidence of stroke [115]. In contradistinction to PLAAO, the application of a left atrial appendage clipping not only effectively resolves the issue of left atrial thrombosis but also achieves electrical disconnection of the left atrium, further decreasing the recurrence of atrial fibrillation [116]. Since no foreign body was detected within the cardiac chamber and the lining of the left atrium appeared smooth, postoperative anticoagulation therapy was deemed unnecessary [117]. The efficacy rate of epicardial LAAO procedures was 92.2%, with no incidence of thromboembolism or serious complications directly attributable to the device during minimally invasive cardiac surgery including cardiac surgeries performed through a minithoracotomy [118]. In conjunction with other cardiac surgical procedures, the clips have been correlated with a reduced incidence of cerebrovascular accidents and has a superior risk-to-benefit ratio [119]. Different shapes and sizes of the LAA can be clipped without the risk of bleeding and tears. Current research initiatives are employing immersive three dimensional (3D) virtual reality technologies to assess the dimensions of the LAA for the purpose of selecting an appropriate atrial clamp and for optimal positioning [120]. Compared with the PLAAO, the surgical procedure involving the application of a left atrial clamp is characterized by precise placement of the clamp, and a consistent, stable and dependable therapeutic outcome (Table 2).

**Table 2.** **Comparison different methods of LAAO**.

Methods of LAAO	Percutaneous left atrial appendage occlusion	Transepicardial left atrial appendage clipping
Advantages	1. The subcutaneous injury was minor.	1. Transepicardial clipping avoids direct contact with blood and no instrumental thrombus formation, minimizing the risk of hemorrhage associated with prolonged anticoagulant therapy.
	2. The procedure is completed in a brief period.	2. Suitable for left atrial appendages of various shapes and opening positions.
	3. The patient recovered quickly after surgery	3. Direct vision is entirely obscured at the base, and the bleeding from the atrial appendage may be minimal.
		4. The endocardium remains consistently intact and exerts a specific effect on reducing left atrial volume.
		5. Reduce the cost of surgery.
Disadvantages	1. The occluder, being in direct contact with the blood, has developed an instrumental thrombus, necessitating long-term anticoagulation therapy.	1. Wound infection.
	2. The unique morphology of the left atrial appendage led to the absence of a suitable occluder in certain patients.	2. Injury of phrenic nerve.
	3. Intrarenal damage, a high rate of bleeding, and the ease of cardiac tamponade.	
	4. Enhance the tension at the heart’s base and ear, leading to weakened sealing or ectopic shedding.	
	5. Treatment costs are higher.	

LAAO, left atrial appendage occlusion.

#### 2.1.4 Surgical Exclusion of the LAA and Corresponding Devices

In 2010, the AtriClip LAAO system became available in the US, offering a epicardium method to occlude the LAA. This procedure allows for direct visualization and can be efficiently used in various cardiac surgical procedures [121]. The AtriClip features two parallel, rigid titanium tubes enveloped by an elastic nitinol spring, which is further encased in a braided polyester sheath [122]. Released at the base of the left atrial appendage, the device blocks blood flow between the appendage and the left atrium, thereby isolating the appendage (https://www.atricure.com/healthcare-professionals/therapies/LAAM/atriclip-exclusion-system) [123]. This procedure aims to prevent the occurrence of thrombosis and stroke by further ligation or resection of the LAA after clipping the LAA [124]. The objective of left atrial appendage clipping can be accomplished through a total sternotomy, a minimally invasive incision, or via total thoracoscopic implantation [125, 126]. A randomized controlled trial that compared three surgical techniques (stapled excision, internal ligation, and surgical excision) revealed an overall failure rate of 57% [99]. Kiankhooy *et al*. [122] conducted a prospective evaluation of 97 patients with AF undergoing cardiac surgery, assessing the outcomes of AtriClip placement. 74 patients received the AtriClip via video-asisted thoracic surgery (VATS), while 23 underwent sternotomy or thoracotomy without the use of TEE. The study focused on thromboembolic events occurring within one year postoperatively. The results demonstrated that the AtriClip achieved excellent rates of successful left atrial appendage closure (LAAC) [122]. In 2016, the AtriClip PRO2, a new device, was introduced in the US and European markets. The primary distinction between the two devices lies in the orientation of the clamp: the ric clamp is positioned perpendicular to the plane of open surgery, whereas the PRO2 clamp aligns parallel with the handle’s direction, facilitating a complete thoracoscopic implantation via the 12 mm port [127]. The handle allows the surgeon to control the angle and opening and closure of the device, enhancing the efficiency and efficacy of LAAC in minimally invasive heart surgeries. The AtriClip Pro V features a dual-spring mechanism, enabling the clip to assume a “V” configuration upon deployment. The distal tip’s closure mechanism enhances the retention of the device during the clamping process [128]. In a canine study, no complications were observed; the atrium was entirely occluded without any instance of device migration [129]. Compared to the LARIAT epicardial occluder, the AtriClip was associated with significantly lower rates of pericardial effusion, postoperative bleeding, stroke, and thrombosis complications [130].

Stapler excision with appendage removal is also a common epicardium surgical exclusion. Wolf *et al*. [27] used BPRF devices for achieving pulmonary vein isolation and surgical stapler for removing the LAA with a minimally invasive beating heart surgery technique. However, the occlusion rate of early LAAO ranged from 41% to 81%, and postoperative transesophageal echocardiography (TEE) revealed a significantly higher rate of LAA recanalization [82, 105]. It is possible that when the staple line was positioned excessively high and the stump surpassed 1 cm in length, it led to the occurrence of further thrombosis. Furthermore, a stapler is believed to be more traumatic than an Atriclip [131]. The thoracoscopic LAA exclusion was subsequently refined through the integration of an automated, highly flexible spike-cutting instrument [132]. A subsequent two-year follow-up study conducted on 201 patients who underwent treatment with endoscopic staplers and ligation rings revealed that the thoracoscopic stapler ring technique resulted in more secure closure of the LAA in patients diagnosed with nonvalvular atrial fibrillation (NVAF) [133]. The efficacy and safety of LAA removal in minimally invasive cardiac surgery (MICS) has also been thoroughly demonstrated. Within the stapler group, there were no reported complications related to LAA [134]. Utilizing a powered surgical stapler for thoracoscopic left atrial ablation in patients with NVAF resulted in AF-related thromboembolic events [135].

The prevalent methods of LAA isolation and occlusion are summarized in Table 3. We strongly recommend surgical exclusion of the LAA for patients who are at a heightened risk of LAA-related thromboembolism. This recommendation applies to individuals who have already undergone cardiac surgery, those who have failed percutaneous exclusion procedures, or patients who are willing to undergo surgery as a standalone procedure. When surgical resection is not an option, suture ligation and stapler techniques are should be considered. Irrespective of the chosen method, all patients must document exclusion of the LAA through intraoperative and subsequent TEE examinations, in order to mitigate the potential risk of delayed LAA thrombosis.

**Table 3.** **Summary of advantages and disadvantages of each LAA exclusion techniques and devices**.

Exclusion method	Devices/techniques	Advantages	Disadvantages	Suitable crowd
Internal ligation	1. Simple suture ligation	1. Inexpensive	1. High recanalization rate	1. Concomitant with other cardiac surgeries or Repeat Heart Surgery.
	2. Single-or-double-layer endocardial suture exclusion.	2. Direct visualization of the endocardial LAA	2. Risk of coronary artery injury	2. Percutaneous (transcatheter) exclusion failure or contraindications.
	3. Purse string suture			
	4. Reinforce running suture			
	5. Modified internal ligation (with compression stitch)			
	6. Biomatrix patch occlusion (alternative ligation)			
Epicardial device-enabled techniques	1. Endoloop snaring	1. Highly effective	1. Epicardial inflammation or adhesion	1. Concomitant cardiac surgery without the incision of the LA or Repeat Heart Surgery.
	2. TigerPaw II system	2. Use in sternotomy, minimally invasive surgery or thoracoscopic surgery	2. Technically demanding	2. Percutaneous (transcatheter) exclusion failure or contraindications.
	3. AtriClip LAA exclusion system (epicardial clipping)			3. Patients with a high risk of bleeding.
	4. Surgical staplers			
Surgical excision	1. Straightforward intra-atrial resection procedure	1. Inexpensive	1. Bleeding	1. Concomitant cardiac surgery without the incision of the LA.
	2. Epicardium surgical resection with suture or scissors	2. Removal of LAA drastically	2. Risk of coronary artery injury	2. Percutaneous (transcatheter) exclusion failure or contraindications.
		3. Simple operation		

### 2.2 Post-Operative Atrial Fibrillation

Post-operative atrial fibrillation (POAF) is a common clinical presentation after cardiac surgery, with an incidence of 20%–40% [136]. It may result in major adverse clinical events, including stroke, perioperative myocardial infarction, and increased mortality within one year post-surgery [137]. A study has shown that the risk of late AF recurrence in patients who have experienced POAF following cardiac surgery is 4 to 8 times greater [138]. POAF episodes that persist for 48 hours are predictive of recurrent episodes of AF [139]. The combination of preoperative left atrial volume index (LAVI) and postoperative interleukin-6 (IL-6) is predictive of POAF. In a study involving 102 patients, it was observed that those who developed POAF exhibited elevated levels of these two indicators [140].

Posterior pericardiotomy (PP) has emerged as another technique to decrease POAF [141]. The primary mechanism by which PP can decrease the incidence of POAF is largely attributed to the reduction of effusions in the posterolateral pericardium [142]. In a randomised, controlled trial, patients undergoing coronary artery, aortic valve, or ascending aorta surgery with or without PP, the incidence of postoperative pericardial effusion was significantly lower in the group that received PP compared to the group that did not undergo any intervention [143]. The incidence of POAF can reach as high as 40% following CABG [144]. Performing PP may reduce the incidence of postoperative pericardial effusion and associated atrial fibrillation by enhancing pericardial drainage following CABG [145]. A meta-analysis encompassing 10 randomized controlled trials (RCTs) demonstrated that posterior pericardiotomy effectively reduces the incidence of atrial fibrillation, pericardial effusion, and shortens hospital stays following CABG. This procedure has been shown to be safe, efficacious, and cost-effective [146]. In addition to performing PP, administering CaCl2 into the primary atrial ganglionic plexus (GP) during CABG can suppress the function of these plexuses and thereby decrease the occurrence of POAF by inducing autonomic neurotoxicity mediated by calcium [147]. A recent pilot feasibility study proposed that administering human placental membrane allografts (HPMA) prior to pericardial closure could offer a novel approach to alleviate POAF after CABG by modulating local inflammation. This method may also reduce the duration of the intensive care unit (ICU) stay and overall hospitalization, ultimately improving patient outcomes [148].

### 2.3 Surgical Biomaterial for Restoring Conduction

While the surgical techniques and related to the interventions described above can alleviate symptoms of AF and decrease complications to a certain degree, it’s important to note that surgical intervention may not be suitable for all patients due to potential contraindications or limitations. In recent years, surgical biomaterial techniques for re-entrant arrhythmia by restoring conduction are gradually being introduced for the treatment of AF.

The prevalent mechanism underlying numerous arrhythmias is reentry, which can occur when non-conductive scar tissue disrupts the autonomic conduction pathway [149]. These arrhythmias are typically managed using antiarrhythmic medications and myocardial ablation, however, both approaches carry the risk of adverse side effects and have limited effectiveness [150, 151]. An evolving technique involves the injection of biomaterial and cells into the intimal myocardium, with the goal of restoring the natural conduction of the myocardium that has been damaged or diseased, preventing scar formation, or enhancing cardiac function [152]. Surgical biomaterial for restoring conduction primarily involves those with enhanced biocompatibility, such as biological electrode materials and tissue engineering brackets, which are utilized for the creation of electrophysiological structures during surgical procedures to modulate atrial conduction.

Biological electrode materials involve the placement of conductive substances on regions with compromised electrical conductivity, enabling the synchronized stimulation of the myocardium, which could theoretically inhibit the development of reentrant circuits [153]. Cardiac patch materials, serving as biological electrode materials, can markedly enhance the transmission efficiency of electrical signals and diminish postoperative complications [151]. Conductive and viscous hydrogels can be readily applied to the heart’s surface as functional patches that mimic the properties of healthy myocardium, without causing adverse fluid leakage, which can integrate with the beating heart within four weeks, significantly enhancing the transmission of electrophysiological signals [154]. Carbon nanotube patches, composed of nanofibrillated cellulose/single-walled carbon nanotube, were evaluated for their conductivity, flexibility, and stretchability when applied to the canine epicardium, with the aim of restoring conduction in areas where activation had been disrupted [155]. A highly conductive cardiac patch has been developed, which integrates bioabsorbable metals and polymers to create a composite material structure. This structure offers mechanical reinforcement and establishes supplementary conductive pathways, synchronizing the excitation and contraction of the heart in patients suffering from myocardial infarction [156].

With the development of regenerative medicine, the use of biomaterial stents in the regeneration of cardiac tissue has gradually increased, especially the application of conductive tissue-engineered scaffolds in myocardial infarction [157]. Previous studies indicated the poly lactic acid/polyaniline (PLA/PANI) conductive nanofibrous sheets with much higher spontaneous beat frequency and extracellular matrix like nanostructure, demonstrating promising potential in cardiac tissue engineering and cardiomyocytes-based 3D bioactuators [158]. The stent used for cardiac tissue engineering is a three-dimensional porous structure made from biocompatible materials, whose main role is to simulate the extracellular matrix of the heart, support the cell growth of cardiac cells, and promote cell adhesion and proliferation, thus forming new bioconductance pathways [159]. Later, the field of tissue engineering witnessed a revolutionary shift in cardiac cell regeneration with the incorporation of carbon-based nanomaterials, which boasted superior variability, electrical conductivity, and mechanical properties [160]. Given that these scaffolds can foster cardiac tissue regeneration and restore conduction function by offering a bioactive and conductive environment, might we consider that they offer novel insights for the treatment of AF? Although tissue-engineered scaffolds show good application potential in laboratory settings, evidence in clinical applications is still limited and larger multicenter clinical trials are needed to verify their safety and efficacy in AF.

## 3. Conclusions

Several surgical techniques and devices, rooted in the Cox-Maze procedure, have emerged, each with varying potential for success. However, the question remains as to whether these therapeutic strategies truly represent the most effective approach for mitigating AF. The majority of study designs pertaining to surgical procedures and associated devices tend to be retrospective in nature, while a substantial portion of prospective studies are frequently confined to a single center. Consequently, it is imperative to exercise caution when drawing conclusions concerning success rates and complication rates, as these factors may be influenced by the limitations inherent in these study designs. POAF has received increasing attention as the most prevalent clinical complication, yet it remains uncertain whether various pathological or drug-mediated mechanisms can be effectively harnessed for the prevention and management of this condition. Surgical biomaterial approaches, which encompass electrical conductivity, compatibility, and mechanical strength, exhibit significant advantages in surgical interventions and have potential for further development. Future research should delve deeper into the long-term safety and efficacy of their biocompatibility, and advance the standardization of their clinical applications. The experts of the European Heart Rhythm Association/European Association of Percutaneous Cardiovascular Intervention have introduced an innovative algorithm. This algorithm is specifically designed to pinpoint those patients who would derive the greatest benefit from undergoing interventional closure of the LAA. It is worth considering whether the surgical approach can also establish an algorithm to evaluate the treatment of AF patients as an intervention. What is the surgical method and devices or biomaterials to choose? To provide a definitive answer to this query, there is a pressing need for more precise and dependable data derived from rigorously designed, prospective randomized trials adhering to standardized protocols.
